# The Treatment of Tuberculosis by the Administration of Urea

**Published:** 1901-12-14

**Authors:** 


### THE TREATMENT OF TUBERCULOSIS BY THE ADMINISTRATION OF UREA.

Among the various methods which have from time to time been advocated for the cure of tuberculosis the administration of urea certainly seems one of the most curious. Yet so far as mere hypothesis goes, Dr. Harper[1] is able at least to give a more or less plausible explanation of the faith that is in him, which is more than can be said for some of the methods which are from time to time put forward for the treatment of this disease. Convinced of the ubiquitous prevalence of the tubercle bacillus, and more especially of its presence in his own immediate surroundings, he asked himself how it was that he escaped, and on inquiring into his family history he found that gout and its allied diseases were very marked in the families of both his parents. Here, then, was the explanation of his immunity. But if gouty salts be so antagonistic to tubercle as to have protected him, even though bacilli were found in the dust of his own consulting room and on the moustaches of his own patients, why, he asked, should not these gouty salts be administered to those who are tuberculous? For the superior resisting power or mode of defence that is a natural endowment in one group of human beings can most likely be produced by artificial means in another group that are lacking in it. Gout—nitrogen—urea. What more natural, then, than to treat tuberculosis by giving urea? And undoubtedly, so far as the comparative immunity of the gouty, the advantages of a freely nitrogenous dietary in the treatment of consumption, and the comparative immunity of carnivorous animals to tuberculosis, are to be relied upon as indications for treatment, there is something to be said for the dictum that "nitrogen and nitrogenous products are the remedy *par excellence* for the tubercle bacillus." Dr. Harper says that for nearly two years and a half he has been prescribing urea for various forms of tuberculosis. This substance, he says, "exerts a specific action on tuberculosis quite as marked as mercury on a syphilitic node, salicylate of sodium on a painful joint in rheumatic fever, or iodide of potassium on bronchial asthma," and he gives a series of cases which certainly are striking enough. He says—and this is certainly a point worth considering in regard to the trustworthiness of any suggested treatment—that those using urea can study its effects on tuberculous diseases like lupus and superficial tuberculous glands, and watch its action. In regard to its actions upon external tuberculosis he is supported by Dr. Buck,[2] who has given details of a case of lupus vulgaris of 12 years' standing which was cured by the administration of urea. Dr. Harper says that in administering this drug a beginning should be made with small doses of from 10 to 15 grains thrice daily, which should be gradually increased up to 40 or 60 grains as a maximum. We cannot but feel that there are many gaps in Dr. Harper's arguments. Yet it must be remembered that our knowledge as to the nature of the processes by which the onslaughts of tubercle are resisted is but small, and that, for the present at least, our prescriptions for the strengthening of this resistance must be largely empirical. The question of the utility of urea ought to be capable of almost immediate decision one way or another.

[1] Lancet, Dec. 7. [2] Practitioner, July 1901.

